# Behavioral Settings are Crucial for Assessing Sensorimotor, Anxiety, and Social Changes in Aging and Spinal Cord Injury

**DOI:** 10.1002/brb3.70686

**Published:** 2025-07-11

**Authors:** Chloé M. Gazard, Nacéra Douich, Eloïse Néel, Patrick Villette, Florence E. Perrin, Yannick N. Gerber

**Affiliations:** ^1^ MMDN, Univ. Montpellier, EPHE, INSERM Montpellier France; ^2^ Innovation Net Tiranges France; ^3^ Institut Universitaire de France (IUF ) Paris France

**Keywords:** experimental protocol design, open field, physiological aging, sexual dimorphism, spinal cord injury

## Abstract

**Introduction:**

For decades, behavioral tests have been used to evaluate functions and dysfunctions of the central nervous system. The open field test is one of the most commonly used methods to assess murine behaviors, including exploration, spontaneous motor activity, and anxiety‐like behaviors. The choice of open field shape can vary across studies, either to address specific research objectives or to simplify data acquisition and analysis.

**Methods:**

In this study, we investigated whether the shape of the open field arena influences mice's behaviors in an age‐ and sex‐dependent manner during physiological aging. We then assessed how the shape of the open field influences male mice's behaviors in an age‐dependent manner following spinal cord injury.

**Results:**

Across physiological aging, we observed that the shape of the open field arena influenced motor activity, anxiety, and social behavior, with some gender differences. Following spinal cord injury, arena shape affected motor activity, tactile sensitivity, and social behavior in male mice, but anxiety‐like behaviors remained unaffected.

**Conclusion:**

Our results highlight that the shape of the arena significantly influences mouse behavior, emphasizing the importance of careful experimental protocol design in behavioral assessments.

## Graphical abstract

We show that the shape of the open field arena significantly affects mouse responses to various behavioral tests, including anxiety, locomotion, social behavior, and tactile sensitivity, in both physiological aging and after spinal cord injury. Therefore, establishing standardized protocols is particularly crucial.

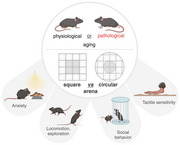

## 1 Introduction

Open field tests rely on the natural tendency of rodents to explore novel environments and their aversion to open spaces. These tests are widely used in neuroscience to evaluate various behaviors, including exploration and anxiety (e.g., time spent in the center versus the periphery, grooming, and responses following food deprivation), locomotor activity, and, with modifications, social interactions. Consequently, open field tests are valuable tools for characterizing physiological functions and dysfunctions, monitoring disease progression, and assessing the effects of therapeutic interventions.

Since their inception in psychology, Hall (1934) highlighted the shape and size of arenas as parameters that “might be expected to yield discrepancies in the rate of habituation” (Hall 1934). Emotionality in rats was investigated using a circular arena with a diameter of 1.2 m (Hall 1934), while discrimination between learning and skill in rats was assessed using a square arena measuring 36 cm on each side and 30 cm in height (Yoshioka 1932).

The choice of a specific open field shape varies among studies, depending on the experimental questions being addressed or the need to facilitate data acquisition and analysis. Square or rectangular arenas allow for the analysis of corner preference, which may reveal specific thigmotactic behaviors. Additionally, when paired with grid lines, these shapes simplify the manual quantification of distance traveled (a method often used before the advent of automatic video tracking systems). Square or rectangular arenas may also be more practical in terms of space utilization compared to circular designs. Conversely, circular arenas create an environment where the natural tendency of rodents to avoid open spaces can be observed without the potential bias introduced by corners. Many additional variables, such as age, sex, and pathophysiological conditions, can influence performance in behavioral tests conducted on mice (Shoji and Miyakawa 2019, Torrisi et al. 2023).

Many studies have documented age‐related behavioral decline (Brito et al. 2023). Recently, a large‐scale analysis of behavioral data revealed that mice exhibited age‐related changes when assessing motor behavior, anxiety, and cognitive performance (Shoji et al. 2016). Another recent report found that grip strength, spontaneous motor activity, and gait velocity declined with age when analyzing performance across different cohorts of mice (Yanai and Endo 2021). Motor performance began to decline as early as 6 months of age, with lower cognitive performance observed later and profound impairments described by 22 months of age (Yanai and Endo 2021). In addition, studies have reported variations in social preference based on age and sex. Males tend to display higher levels of social preference than females (Baumgartner et al. 2023). These levels also increased with age in both sexes (Baumgartner et al. 2023).

Traumatic spinal cord injury (SCI) results in the loss of sensorimotor and autonomic functions, with the severity depending on the extent of the lesion and its rostrocaudal level. Beyond the direct consequences at the site of the injury in the spinal cord, SCI also induces profound and widespread alterations in the brain. In adult rats with incomplete cervical SCI, studies have shown a decrease in the capacity for neurogenesis within the subventricular zone (SVZ) and the subgranular zone (SGZ) (Felix et al. 2012). Furthermore, during the chronic phase, SVZ neurogenesis returns to its basal level, whereas SGZ neurogenesis remains 40% lower than in control animals (Felix et al. 2012). Additionally, in mice, microglial activation within the cortex and hippocampus has been demonstrated following SCI, with a correlation observed between the severity of the lesion and the intensity of cognitive, behavioral, and depressive disorders (Wu et al. 2016). This supraspinal neuroinflammation contributes to neuronal loss and alterations in hippocampal neurogenesis.

In this study, we examined whether the shape of the open field arena influences behaviors in an age‐dependent manner, both during physiological aging and after SCI in CX3CR1^+/eGFP^ mice. During physiological aging, we found (1) a sex‐dependent effect of arena shape on spontaneous motor activity, (2) no effect on tactile sensitivity for both sexes, (3) an effect of arena shape on latency to eat and eating duration regardless of sex, and (4) an influence of arena shape on social behavior in both young and aged mice. After SCI in male mice, the arena shape influenced motor activity, tactile sensitivity, and social behavior but had no effect on anxiety.

## 2 Methods

### 2.1 Mice

Heterozygous transgenic male and female mice expressing enhanced green fluorescent protein (eGFP) in central nervous system (CNS) resident microglia and circulating peripheral monocytes (CX3CR1^+/eGFP^) were used in all experiments. These mice were obtained from Dr. Dan Littman (Howard Hughes Medical Institute, Skirball Institute, NYU Medical Center, New York, NY, USA) (Jung et al. 2000). We used this strain because all SCI‐related studies in our laboratory are conducted on the CX3CR1^+/eGFP^ strain. These mice express eGFP under the control of the Cx3cr1 promoter. The transgenic line was maintained on a C57BL/6 background (The Jackson Laboratory, Bar Harbor, ME, USA, RRID:IMSR_JAX:005582). Mice were housed under controlled hygrometry and temperature conditions, with a 12‐h light/dark cycle and continuous access to food and water. Four different groups of mice were used (see Table 1 for details).

**TABLE 1:** Mice groups and behavioral assessments in our study.

Groups and the number of mice	Condition	Open field (motor and anxiety)	Open field (tactile)	NSF test	Social
Group 1 13 M, 14 F	Physiological aging	3, 12, 15, and 18‐months‐old mice	3, 12, 15, and 18 months old	3, 12, 15, and 18 months old	
Group 2 8 M, 5 F	Physiological aging				4 months old
Group 3 6 M, 6 F	Physiological aging				12 months old
Group 4 7 M	SCI at 3 months of age	Prior SCI, 1, 2, 4, and 6 wpi and 6, 8, 10, and 12 mpi	Prior SCI, 5 wpi and 6, 8, and 12 mpi	Prior SCI, 6 wpi and 6, 8, 10, and 12 mpi	12 mpi

Abbreviations: F, female; M, male; mpi, month post injury; NSF, novelty‐suppressed feeding; wpi, Week post injury.

### 2.2 Spinal Cord Injury

At 3 months of age, male mice were anesthetized with isoflurane gas (3% for induction and 1.5% for maintenance during the surgical procedure). A laminectomy was performed to remove the posterior arch of the thoracic 9 (T9) vertebra, followed by a lateral hemisection of the spinal cord under a microscope (Leica Microsystems, RRID:SCR_027099) using an ophthalmic blade (10315–12, Fine Science Tools, Heidelberg, Germany), as previously described (Poulen, Bartolami, et al. 2021).

The muscles and skin layers were sutured, and we monitored the mice during the recovery period following the injury. After recovery, the mice were returned to their home cages. Bladders were manually emptied twice daily until sphincter control was regained. Body weights were recorded prior to surgery and then monitored monthly throughout the study. Seven male mice were used in this experiment (Group 4; see Table 1). Male mice were selected for this study because epidemiological data indicate that adult males have a higher incidence of traumatic SCI compared to females, with a reported male‐to‐female ratio of at least 2:1 (WHO 2013).

### 2.3 Behavioral Tests

**FIGURE 1:**
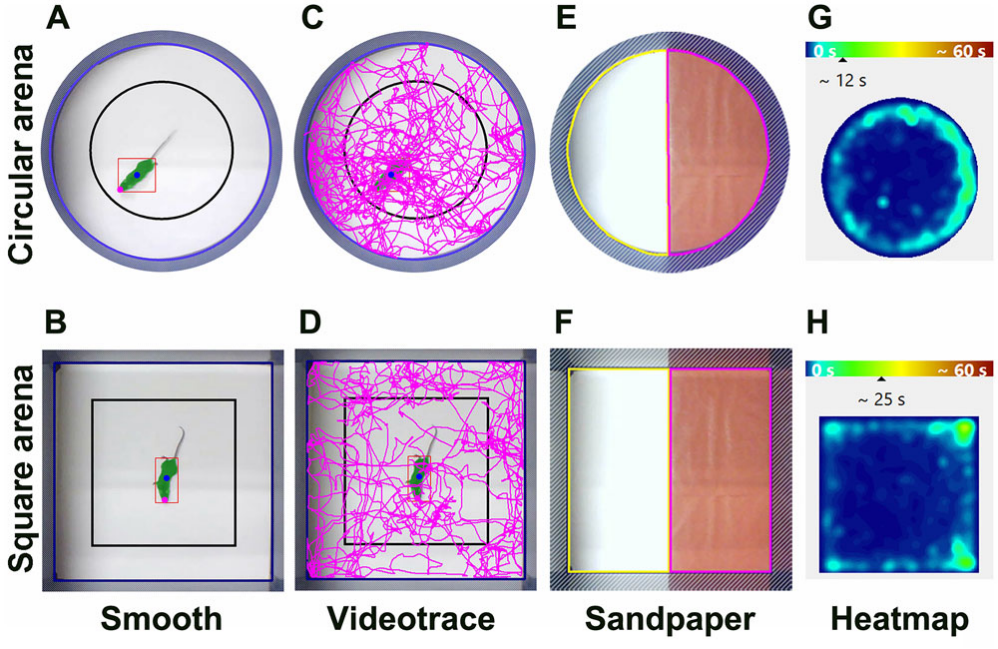
Open field experimental setup, videotracking, and heatmaps. Representative examples of video‐based mouse detection using EthoTrack software in both circular (A, C, E, and G) and square (B, D, F, and H) arenas. Spontaneous motor activity during an 8‐min session is represented by a pink trajectory line (C and D). Regions for analysis are defined as follows: blue line = arena (A–D); black line = center of the arena (A–D). The detection system captures the entire mouse body (highlighted in green and surrounded by a red dashed line), along with its head (pink dot) and its barycenter (blue dot). The experimental setup for tactile sensitivity assessment using sandpaper is shown for the circular (E) and square (F) arenas. Regions for analysis are defined as follows: yellow = smooth surface; pink = sandpaper surface. Examples of heatmaps illustrating the mean duration spent per zone and per group in circular (G) and square (H) arenas with sandpaper‐grounded floors on the right side of the arena.

Behavioral tests included habituation to the experimenter, the novel environment, and the apparatus to minimize stress‐induced bias. Recording sessions were conducted in two arena types: a circular arena (Figure 1A,C,E,G) with a diameter of 45 cm and a square arena measuring 45 cm × 45 cm (Figure 1B,D,F, H). A camera was positioned above each arena for recording. EthoTrack video tracking software (Innovation Net, Tiranges, France, RRID:SCR_027105) was used for recording and analyzing all tests. Both the circular and square arenas featured a white floor surrounded by blue walls (height 33.5 cm) (Figure 1A–D). The mean detection accuracy across all animals and tests was 99.74%, with a mean head detection accuracy of 98.86% specifically for the novelty‐suppressed feeding test.

#### 2.3.1 Spontaneous Motor Activity, Exploration, and Anxiety

Spontaneous motor activity and anxiety levels were assessed using the open field test. Mice were individually placed in either an empty circular or a square test arena. After a 30‐s acclimatization period, their spontaneous locomotor activity was recorded for 8 min. The total distance traveled (m) by each mouse during the session was measured, along with the time spent (s) in the periphery and the center of the arenas (Figure 1C,D). In both the circular and square arenas, the center area accounted for approximately 42% of the total area (Figure 1A,B).

#### 2.3.2 Tactile Sensitivity

Tactile sensitivity was assessed using the same arenas. Half of the arena floor was covered with smooth paper, while the other half was covered with sandpaper (granularity of 50), as previously described (Figure 1E,F) (Noristani, They, et al. 2018, Bringuier et al. 2023). Quantifications were performed during 8‐min recorded sessions, following a 30‐s acclimatization period. The time spent in each zone was measured for each session.

#### 2.3.3 Novelty‐Suppressed Feeding Test

This test was used to assess anxiety‐related behavior (Bodnoff et al. 1988). Following 24 h of food deprivation, the animals were given the opportunity to choose between eating food pellets placed in the center of either a circular or square arena (an area corresponding to 2.3% of the total arena) or staying in the periphery. The recording session lasted for 5 min. We quantified the latency to eat, defined as the time spent by the animal before entering the center to eat, as well as the time spent eating.

#### 2.3.4 Social Behavior

This test was used to assess social interaction (You et al. 2019). Two identical cage chambers were placed within an arena, which could be either square or circular. The test consisted of two steps. For habituation to the environment, mice were placed in the arena (either square or circular) with two empty chambers for 10 min, 24 h prior to the social behavior assessment (see insets in Figure 4), without recording their activity. On the following day, social interaction testing included two consecutive 8‐min sessions. To eliminate preference bias, the mice were placed in an arena containing two empty chambers during the first video‐recorded session. In the second session, a stranger mouse (an unfamiliar mouse of the same sex, age, and strain) was introduced into one of the chambers, while the other chamber remained empty. We quantified the time spent in the vicinity of each chamber during both sessions.

### 2.4 Statistics

Two‐way repeated measures ANOVA followed by Bonferroni post hoc tests was used for behavioral comparisons between arena shapes or sex over time in physiological aging or after SCI. One‐way ANOVA followed by Dunnett post hoc tests was applied to compare the evolution of a given parameter over time after SCI. Unpaired *t*‐tests with Welch's correction were used for social behavior comparisons. Significance was set at *p* ≤ 0.05. Results are presented as mean ± standard error of the mean (SEM). All statistical analyses were performed using GraphPad Prism v5.0 software (GraphPad Software, Boston, USA, RRID:SCR_002798).

## 3 Results

To investigate potential differences in behavioral responses based on the shape of the open field arena, we conducted a longitudinal analysis of two cohorts of mice (males and females) at various ages (3, 12, 15, and 18 months) under physiological conditions. We directly compared data obtained from circular and square open fields across several parameters, focusing on sensorimotor responses, anxiety‐related behaviors, and social interactions. Subsequently, we evaluated these same parameters in both arena shapes within the pathological context of SCI.

### 3.1 Open Field Arena Shape Influences Spontaneous Motor Activity in Females Only, but Not Tactile Sensitivity During Physiological Aging

**FIGURE 2:**
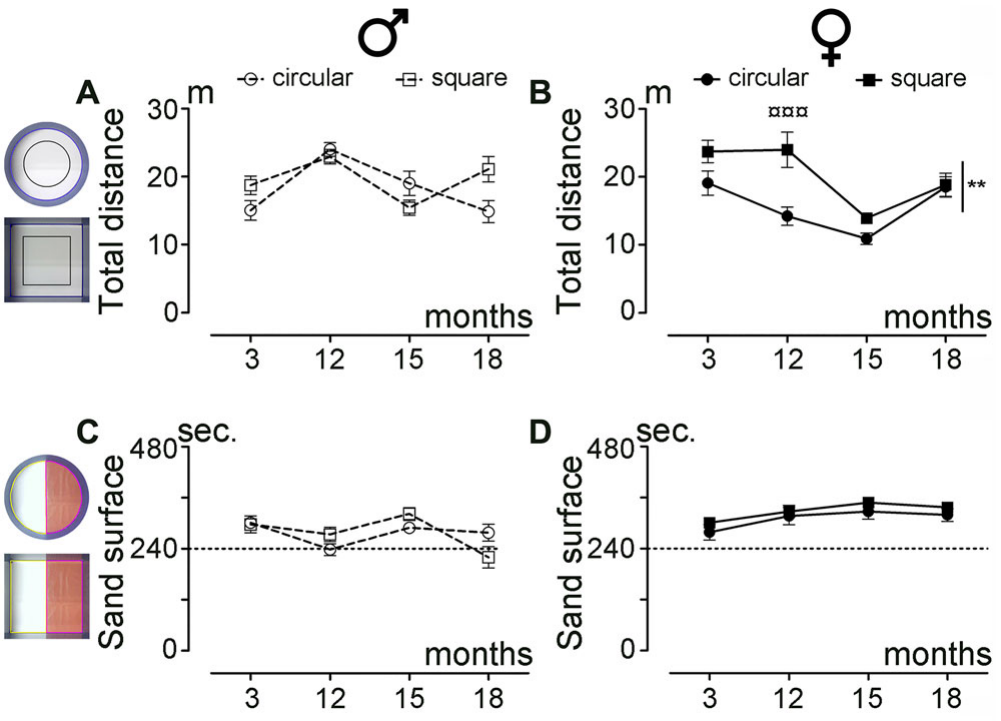
Influence of the open field shape on sensorimotor activity over physiological aging. Analysis of the covered distance (A, B) in a circular or square arena by male (A) and female (B) mice over aging. Analysis of the time spent on the sand surface (C, D) in a circular or square arena by male (C) and female (D) mice over aging. Results are presented as mean ± SEM per time point for males (empty circles or squares with dashed lines (A, C) and for females (plain circles or squares with solid lines [B, D]). Statistics: two‐way ANOVA (**p ≤ 0.01), followed by Bonferroni post hoc test (¤¤¤*p* ≤ 0.001). Number of mice: 13 males and 14 females per experiment and per age. The corresponding circular or square arena for each recorded session is depicted on the left side. For spontaneous motor activity (A, B), regions for analysis are defined as follows: blue line = arena; black line = center of the arena; for tactile sensitivity assessment, regions are defined as follows (C–D): yellow = smooth surface; pink = sandpaper surface.

Spontaneous motor activity was first assessed by analyzing the distance covered by the same animals in both types of arenas (Figure 2A,B). Overall, male mice covered similar distances in both the circular and square arenas (Figure 2A; *p* = 0.3352). In contrast, female mice covered a greater distance in the square arena compared to the circular arena (Figure 2B; *p* = 0.0023). Additionally, we observed no difference between sexes for this parameter in either the circular arena (Figure S1A; *p* = 0.0581) or the square arena (Figure S1B; *p* = 0.6833). For the tactile sensitivity assessment over aging, mice were placed in the same open field, divided into two sections, one covered with sandpaper (pink delineation) and the other with smooth paper (yellow delineation) (Figure 1E,F). Both sexes spent more time on the sandpaper than on the smooth area (Figure 1G,H) (mean for males: 277 out of 480 s, or 58%; mean for females: 319 out of 480 s, or 67%) (Figure 2C,D). Direct comparison between sexes revealed that females spent more time on the sandpaper in both the circular arena (Figure S1C; *p* = 0.0396) and the square arena (Figure S1D; *p* = 0.003) than males. However, no difference was observed between the shapes of the arena in either sex (Figure 2C; *p* = 0.8976 and D, *p* = 0.2425).

In summary, while female mice showed differences in spontaneous motor activity based on arena shape, no such differences were observed in males. Both sexes spent more time on sandpaper than smooth paper, with no significant effect of arena shape on this behavior.

### 3.2 Open Field Arena Shape Influences Spontaneous Anxiety Levels During Physiological Aging

**FIGURE 3:**
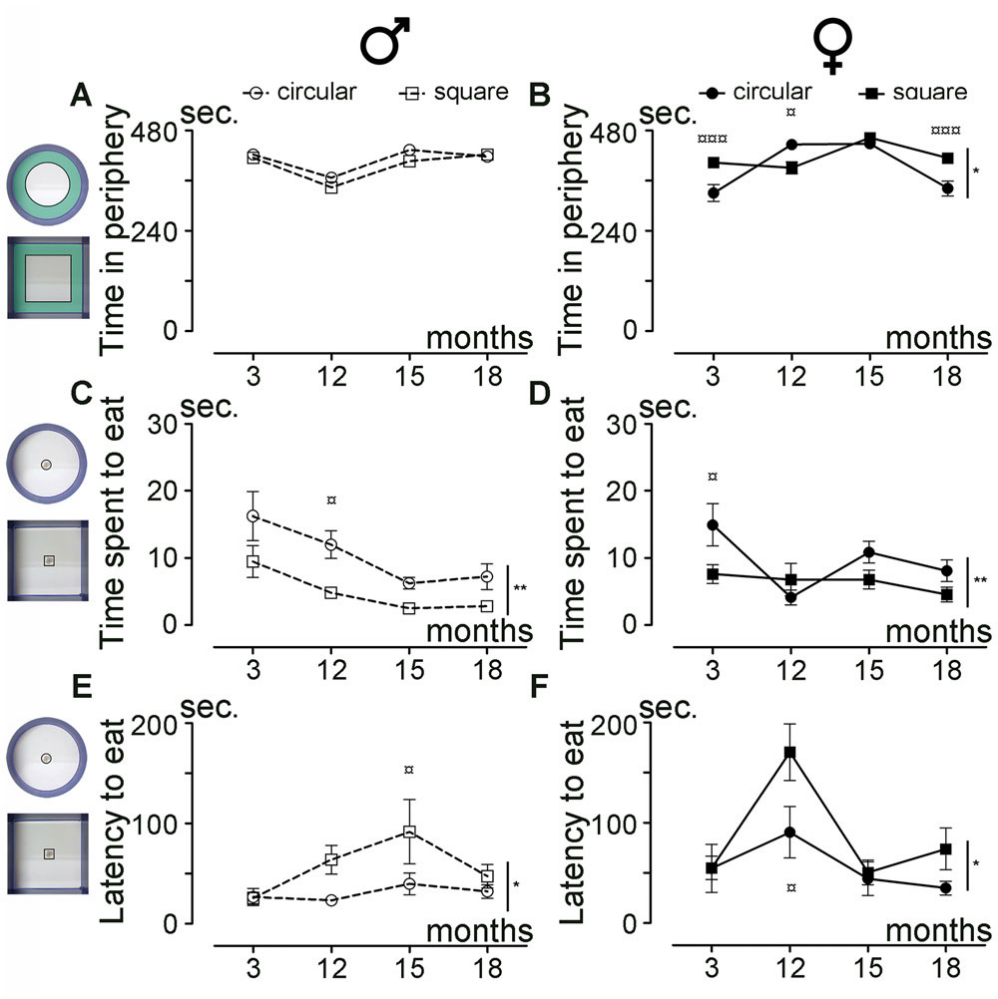
Influence of the open field shape on anxiety over physiological aging. Analysis of the time spent in the periphery (A, B) in a circular or square arena by male (A) and female (B) mice over aging. Analysis of the time spent eating (C, D) in a circular or square arena by male (C) and female (D) mice over aging. Analysis of the latency to eat in a circular or square arena by male (E) and female (F) mice over aging. Results are expressed as mean ± SEM per time point for males (empty circles or squares with dashed lines [A, C, and E]) and for females (plain circles or squares with solid lines [B, D, and F]). Statistics: two‐way ANOVA (*p ≤ 0.05; **p ≤ 0.01), followed by Bonferroni post hoc test (¤*p* ≤ 0.05; ¤¤¤*p* ≤ 0.001). Number of mice: 13 males and 14 females per experiment and per age. The corresponding circular or square arena for each recorded session is depicted on the left side of the figure. For the time spent in the periphery, the region for analysis is highlighted in green (A, B). For the novelty suppressed feeding test (NFST), regions for analysis are defined as follows: blue line = arena; black line = food pellets (C–F).

Next, we investigated how the shape of the open field arena affects anxiety levels across aging in both sexes (3, 12, 15, and 18 months of age). Using recordings of spontaneous motor activity, we analyzed the time spent exploring the periphery of the arena (highlighted in green in the insets, Figure 3A) as an indicator of anxiety levels. Overall, male mice spent a similar amount of time in the periphery in both the circular and square arenas (Figure 3A; *p* = 0.0807). In contrast, female mice spent significantly different amounts of time in the periphery depending on the arena shape (Figure 3B; *p* = 0.0128). Direct comparison between sexes revealed that females spent more time than males in the periphery in the square arena (Figure S2B; *p* = 0.0121) but not in the circular arena (Figure S2A; *p* = 0.0668). Furthermore, we investigated how the shape of the open field arena affects anxiety using the novelty suppressed feeding test (NSFT), analyzing the duration of eating and the latency to eat food pellets placed in the center of the arena (insets, Figure 3C,E) following 24 h of food deprivation. Male (Figure 3C; *p* = 0.0013) and female (Figure 3D; *p* = 0.0047) mice spent more time eating in the circular than in the square arena. This was associated with a longer latency to eat in the square arena than in the circular arena for both sexes (Figure 3E; *p* = 0.0105; Figure 3D; *p* = 0.0332). Direct comparison between sexes revealed no difference in both arena shapes for the time to eat (Figure S2C; *p* = 0.5273; Figure S2D; *p* = 0.1646). However, analysis of the latency to eat between male and female mice across aging revealed that females consistently took longer than males to access the food pellets, irrespective of arena shape (Figure S2E; circular arena *p* = 0.0360; Figure S2F; square arena *p* = 0.0248).

In conclusion, the shape of the arena affects anxiety levels across aging, with female mice spending more time in the periphery of the square arena compared to the circular arena. Additionally, mice exhibited a longer latency to eat in the square arena, accompanied by a shorter eating duration, regardless of sex.

### 3.3 Open Field Arena Shape Influences Social Behavior in Young and Aged Mice

**FIGURE 4:**
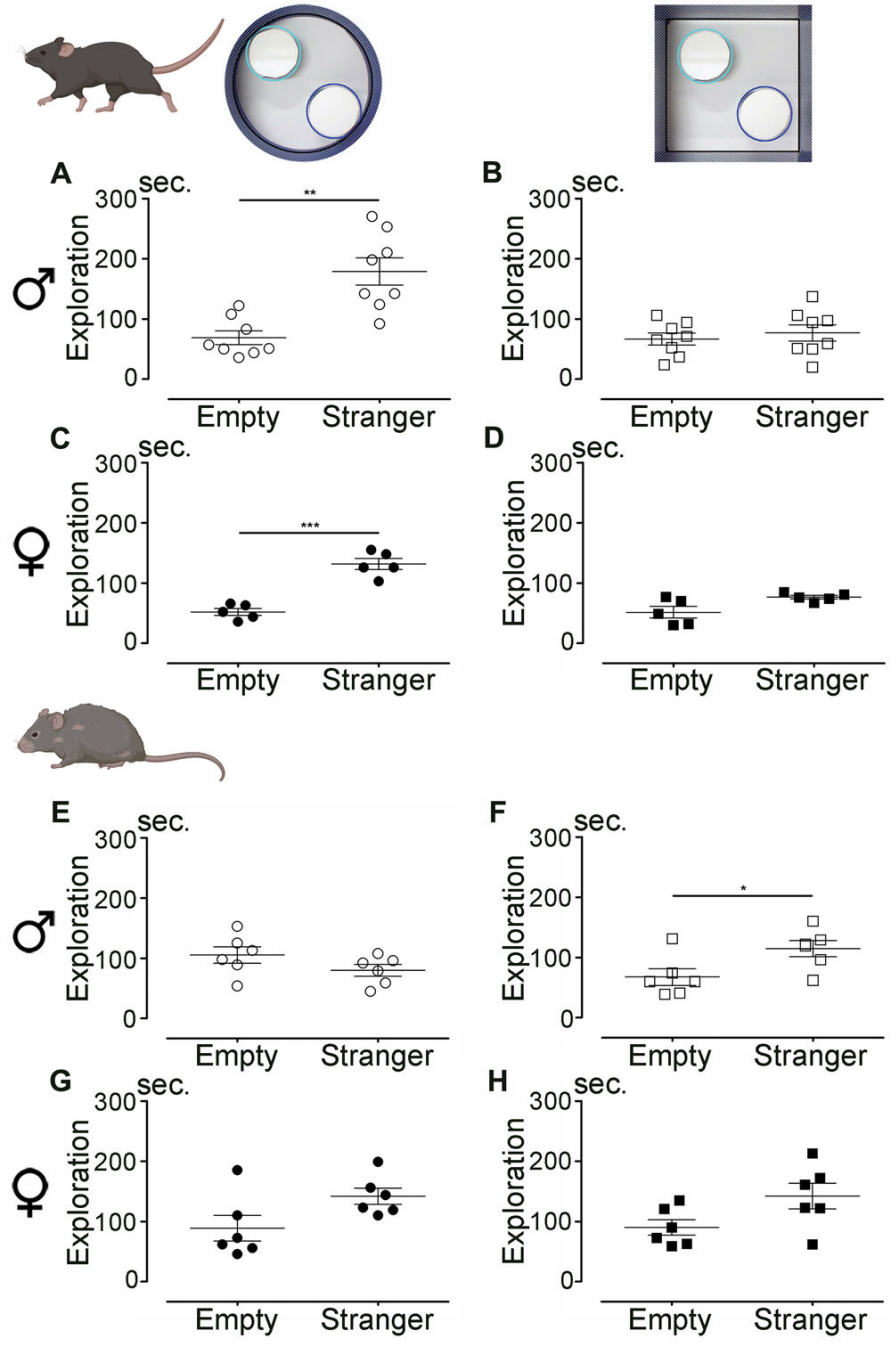
Influence of the open field shape on social behavior in young and aged mice. Analysis of the time spent by 4‐month‐old (A–D) and 12‐month‐old (E–H) male (A, B, and E, F) and female (C, D, and G, H) mice in the close vicinity of an empty area or in the close vicinity of a stranger mouse in a circular (A, C, E, and G) or square (B, D, F, and H) arena. Results are expressed as mean ± SEM for males (clear circles or squares) and for females (plain circles or squares). Statistics: *t*‐test with Welch's correction (*p ≤ 0.05; **p ≤ 0.01; ***p ≤ 0.001). The corresponding arena used for each social behavior recorded sessions is presented at the top of the panel. Regions for analysis are defined as follows: dark line = arena; light blue line = zone 1; dark blue line = zone 2. The empty area and the area with the stranger are swapped between each animal tested. Number of mice: eight males and five females (at 4 months old) and six of both sexes (at 12 months old).

We aimed to investigate whether the shape of the open field influences social behavior in young (4 months old) and aged (12 months old) mice of both sexes. To begin, we assessed social behavior in young mice (Figure 4A–D). During habituation trials, no position preference was observed, as all mice spent equal time near each empty chamber (experimental setup shown in the insets of Figure 4A,B).

During the social interaction assessment in the circular arena (Figure 4A), young males spent significantly more time near the chamber containing the stranger mouse compared to the empty chamber (*p* = 0.0014). However, in the square arena, they spent equal amounts of time near both chambers (Figure 4B; *p* = 0.5559). Similar results were observed in young females, who spent more time near the stranger in the circular arena (Figure 4C; *p* = 0.0003) but showed no preference in the square arena (Figure 4D; *p* = 0.0681). Social behavior was then evaluated in a cohort of aged mice (Figure 4E–H). Habituation trials revealed no position preference. In aged males, no significant difference was observed in the circular arena, with equal time spent near the stranger and the empty chamber (Figure 4E; *p* = 0.1646). However, in the square arena, aged males spent more time near the stranger (Figure 4F; *p* = 0.0370). Aged females showed no significant differences in social interaction in either the circular (Figure 4G; *p* = 0.0673) or square arena (Figure 4H; *p* = 0.0691).

In summary, the shape of the open field influences social behavior in young mice. Both males and females preferred the chamber containing the stranger mouse in the circular arena but showed no preference in the square arena. In aged mice, males displayed the opposite pattern, preferring the stranger in the square arena but not in the circular one, while females showed no significant social preference in either arena shape.

### 3.4 Open Field Arena Shape Influences on Motricity, Tactile Sensitivity, and Social Behavior After SCI in Male Mice

We next aimed to determine whether the shape of the open field could influence behavioral parameters in a pathological context. To this end, we conducted a longitudinal analysis of a cohort of male mice subjected to SCI at 3 months of age. Behavioral assessments were performed early after injury (1, 2, 4, and 6 weeks post‐injury [wpi]) and at later time points during the chronic phase (6, 8, 10, and 12 months post‐injury [mpi]).

**FIGURE 5:**
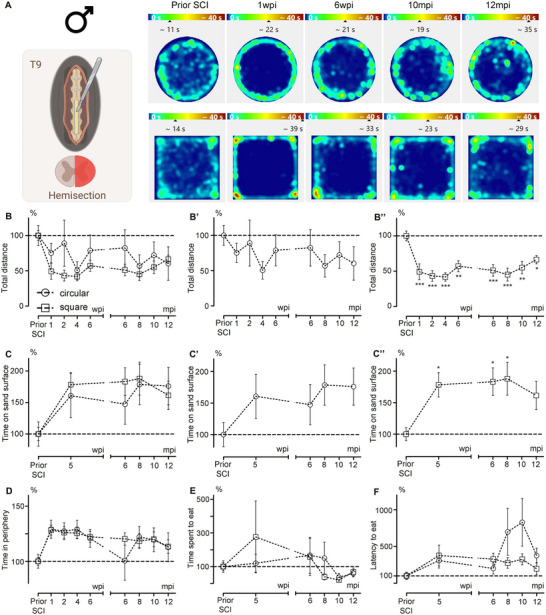
Influence of the open field shape on sensorimotor activity and anxiety after SCI in male mice over aging. Sensorimotor activity using the open field after spinal cord injury (A–C). Schematic view of a lateral SCI. Heatmaps of the mean duration spent per zone and per group in a circular or square arena prior to SCI and at 1 and 6 wpi and 10 and 12 mpi (A). Analysis of the covered distance by male mice in a circular and square open field arena at acute and chronic stages after SCI (B, Bʹ, and B″). Analysis of the time spent on the sand surface by male mice in a circular and square open field arena at acute and chronic stages after SCI (C, Cʹ, and C″). Anxiety assessment using the open‐field after SCI (D–F). Analysis of the time spent in the periphery in a circular or square open‐field arena at acute and chronic stages after SCI (D). Analysis of the time spent eating in a circular or square open field arena after SCI (E). Analysis of the latency to eat in a circular or square open field arena after SCI (F). Results are expressed as a percentage of the value obtained by each animal before SCI and as mean ± SEM per time point for each arena (clear circles or squares). Statistics: two‐way ANOVA (ns), followed by Bonferroni post hoc test (B–F); and one‐way ANOVA, followed by Dunnett post hoc test (*p ≤ 0.05; **p ≤ 0.01; ***p ≤ 0.001; Bʹ, B″, Cʹ, and C″). Number of mice: seven males per time‐point.

Heatmaps of the mean time spent per zone showed that spontaneous motor activity was affected both short term (1 and 6 wpi) and long term (10 and 12 mpi) compared to profiles obtained prior to SCI (Figure 5A).

First, we analyzed the total distance covered by injured mice in circular and square arenas (Figure 5B). A direct comparison of the arenas using a two‐way ANOVA revealed no effect of arena shape on the assessment of recovery after injury (Figure 5B; *p* = 0.3239). However, a one‐way ANOVA analyzing recovery over time within each group showed that distances covered in the circular arena remained unchanged at all time points compared to pre‐SCI values (Figure 5Bʹ; *p* = 0.7886). In contrast, the distance covered in the square arena was significantly reduced at all time points post‐injury (Figure 5B″; *p* = 0.0001). Next, we evaluated tactile sensitivity after injury in both arena types during the early stage (5 wpi) and at more chronic stages (6, 8, and 12 mpi) (Figure 5C). A direct comparison of the arenas using a two‐way ANOVA revealed no significant differences between the circular and square arenas (Figure 5C; *p* = 0.7138). However, a one‐way ANOVA showed no change in the time spent on the sand surface before and after SCI in the circular arena (Figure 5Cʹ; *p* = 0.3599). In contrast, the time spent on the rough surface increased at all time points after injury (except at 12 mpi) in the square arena (Figure 5C″; *p* = 0.0319).

Then, we assessed the level of anxiety using three different parameters: the time spent in the periphery during a regular open field session (Figure 5D), as well as the time spent eating (Figure 5E) and the latency to eat (Figure 5F) during NFST sessions. The analysis of all three parameters showed no influence of the arena shape (Figure 5D,F; D, *p* = 0.08783; E, *p* = 0.9499; F, *p* = 0.2553).

**FIGURE 6:**
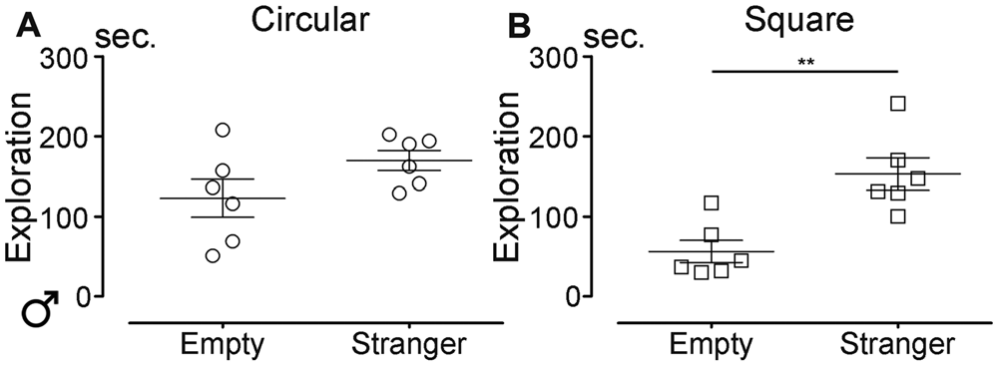
Influence of the shape of the open field on social behavior at the chronic stage after SCI. Analysis of the time spent by male mice in the close vicinity of an empty area or in the close vicinity of a stranger mouse in a circular (A) or square (B) arena at 12 months after SCI. Results are presented as mean ± SEM for each arena (clear circles or squares). Statistics: *t*‐test with Welch's correction (**p ≤ 0.01). Number of mice: six males per time‐point.

Lastly, we assessed social behavior in the cohort of injured mice at the chronic stage (12 mpi). Habituation trials revealed no position preference. At the chronic stage following SCI, injured male mice spent equal amounts of time next to the chamber containing the stranger mouse and the empty one in the circular arena (Figure 6A; *p* = 0.1221). In contrast, in the square arena, male mice spent more time next to the stranger than next to the empty chamber (Figure 6B; *p* = 0.0041). Of note, when comparing social behavior under physiological conditions in aged males (12 mpi) with behavior at the chronic stage after SCI (injury at 3 months of age and behavioral testing at 15 mpi), we found that the shape of the open field influenced the outcomes. In the circular arena, neither uninjured nor injured mice showed a clear preference for interacting with the stranger mouse (Figure S3A,C). By contrast, in the square arena, both uninjured and injured mice spent significantly more time near the chamber containing the stranger mouse compared to the empty one (Figure S3B,D). Interestingly, SCI increased the time spent near the stranger mouse in the circular arena (Figure S3A), whereas this effect was not observed in the square arena (Figure S3B).

Overall, the shape of the open field influenced motor activity, tactile sensitivity, and social behavior in male mice after SCI, while anxiety‐like behaviors were similar across both arena shapes.

## 4 Discussion

In our study, we showed that, in the context of physiological aging, CX3CR1^+/eGFP^ mice exhibited differential responses to the shape of the open field arena in terms of spontaneous motor activity and social behavior, with a notable sexual dimorphism. The shape of the arena affected results obtained with the NSFT (latency to eat and eating duration) but had no impact on tactile sensitivity. In a pathological condition, male mice with SCI displayed differential responses to arena shape in motor activity, tactile sensitivity, and social behavior, but not in anxiety.

We utilized CX3CR1^+/eGFP^ mice due to their relevance in our SCI experiments. We have extensive behavioral data from previous studies (Gerber et al. 2018, Noristani, Saint‐Martin, et al. 2018, Poulen, Aloy, et al. 2021, Poulen, Bartolami, et al. 2021) and several ongoing SCI experiments using these mice. The CX3CR1^+/eGFP^ mice are on a C57BL/6 background and express eGFP under the control of the CX3CR1 promoter, a chemokine receptor found on myeloid cells (Jung et al. 2000). In these heterozygous mice, the CX3CR1 gene is replaced by the eGFP gene. Studies by Piirainen et al. (2021) found no significant behavioral differences between CX3CR1^+/eGFP^ and CX3CR1^+/+^ mice before and after mild chronic restraint stress (Piirainen et al. 2021). Our previous data also show no motor function differences between these mouse groups (Noristani, They et al. 2018), both in uninjured (Figure S4A,C) and SCI conditions (Figure S4B,D). However, caution is advised, as these findings may not be applicable to all mouse strains due to potential variability in responses across different strains in various behavioral tests.

Our data are consistent with a previous study in which several mouse strains (129S1 and F1 hybrids [NMRIS1, BCS1]) were exposed to circular and square open‐field arenas. In that study, mice's horizontal activity was sensitive to the shape of the open field arena, in contrast to their exploratory behavior (Kalueff et al. 2006).

To our knowledge, only two studies have analyzed the influence of open field shape on behavioral responses. On the 129S1 background, both sexes exhibited differential sensitivity to the shape of the open field arena in terms of their horizontal activity. In the same study, female knockout mice lacking functional vitamin D receptors on the 129S1 background, known for their abnormal anxiety and activity behavior, exhibited similar activity levels in both types of open field shapes (Kalueff et al. 2006). Therefore, mice with the same genetic background may exhibit varying sensitivity to the shape of the open field arena under both physiological and pathological conditions. This aligns with our findings, where the shape of the open field influenced anxiety‐like behavior during physiological aging but not after SCI. Furthermore, comparisons of open field tests conducted in circular and square arenas in rats also revealed differences in the intensity of horizontal activity (Grabovskaya and Salyha 2014).

Interestingly, we observed that social behavior was influenced by the shape of the open‐field arena following SCI. This finding may suggest distant supraspinal consequences of SCI. Indeed, morphological and functional magnetic resonance imaging studies have shown a decrease in the volume of the primary somatosensory and motor cortices in patients with SCI (Jurkiewicz et al. 2006, Freund et al. 2011), which is associated with depression and anxiety (for a review, see Budd et al. 2022). Additionally, in rats, SCI resulted in changes to neurogenesis in the olfactory bulb and hippocampal dentate gyrus, as well as gliogenesis in the dorsal vagal complex of the hindbrain (Felix et al. 2012).

In conclusion, our study underscores the critical importance of carefully controlling experimental parameters in behavioral studies to minimize biases. As previously reported (Lonjon et al. 2009), establishing standardized protocols is particularly crucial, as it enables accurate comparisons across different experiments and laboratories. Neglecting such controls in preclinical studies can inadvertently inflate (or decrease) the perceived efficacy of a drug, but also non‐pharmacological therapeutic strategies, thereby compromising the validity of subsequent clinical trials.

## Author Contributions

**Chloé M. Gazard**: methodology, investigation, data curation, formal analysis. **Nacéra Douich**: data curation, formal analysis. **Eloïse Neel**: data curation, formal analysis. **Patrick Villette**: software. **Florence E. Perrin**: conceptualization, funding acquisition, validation, writing–review and editing, supervision, project administration. **Yannick N. Gerber**: conceptualization, supervision, investigation, formal analysis, data curation.

## Ethics Statement

Studies were approved by the local ethics committee (n°36), by the Veterinary Services Department of Hérault, and by the French Ministry of Higher Education and Research (authorization n°34118). Experiments followed the European legislative, administrative, and statutory measures (EU/Directive/2010/63) and the Animal Research: Reporting of In Vivo Experiments (ARRIVE) guidelines. All efforts were made to reduce the number of animals and their suffering.

## Conflicts of Interest

The authors declare no conflicts of interest.

## Peer Review

The peer review history for this article is available at https://publons.com/publon/10.1002/brb3.70686

## Supporting information




**Supporting Figure 1**: Sex‐dependent sensorimotor activity in male and female mice over physiological aging. Analysis of the covered distance (A, B) by male and female mice in circular (A) and square (B) open‐field arenas over aging. Analysis of the time spent on the sand surface (C, D) by both sexes in a circular (C) and square (D) arena over aging. Results are presented as mean ± SEM per time point for males (empty circles [A] or squares [B] with dashed lines) and for females (plain circles [A] or squares [B] with solid lines). Statistics: two‐way repeated‐measure analysis of variance (ANOVA) (*p ≤ 0.05; ***p ≤ 0.001), followed by Bonferroni post hoc test (¤¤*p* ≤ 0.01; ¤¤¤*p* ≤ 0.001). Number of mice: 13 males and 14 females per experiment and per age.

**Supporting Figure 2**: Sex‐dependent anxiety in male and female mice over physiological aging. Analysis of the time spent in the periphery (A, B) for both sexes in a circular (A) and square (B) open‐field arenas over aging. Analysis of the time spent to eat (C, D) by male and female mice in a circular (C) and square (D) arenas over aging. Analysis of the latency to eat for both sexes in a circular (E) and square (F) arenas over aging. Results are presented as mean ± SEM per time point for males (empty circles (A, C, and E) or squares (B, D, and F) with dashed line) and for females (plain circles (A, C, and E) or squares (B, D, and F) with solid line). Statistics: two‐way ANOVA (*p≤0.05), followed by Bonferroni post hoc test (¤*p* ≤ 0.05; ¤¤*p* ≤ 0.01; ¤¤¤*p* ≤ 0.001). Number of mice: 13 males and 14 females per experiment and per age.

**Supporting Figure 3**: Influence of the shape of the open field on social behavior in old male in physiological context and at chronic stage after SCI Analysis of the time spent by male mice in the close vicinity of an empty area or a stranger mouse in a circular (A) or square (B) arena at 12 months of age, under physiological conditions, and at 12 months post‐injury (i.e, 15 months of age). Representative heatmaps showing time spent in the circular (C) or square (D) arena during the social interaction test in both experimental conditions. The location of the stranger mouse is indicated by “S” on the heatmaps. Results are presented as mean ± SEM for each arena (uninjured mice are represented with clear circles or squares, and injured animals are represented with cross‐filled symbols). Statistics: Unpaired *t*‐test with Welch's correction (*p ≤ 0.05; **p ≤ 0.01; ***p ≤ 0.001; ns: nonsignificant). Number of mice: 6 males per group.

**Supporting Figure 4**: Female CX3CR1 +/+ and CX3CR1 +/eGFP mice display identical open field motor performance under physiological conditions and SCI (A) Analysis of the distance covered in 10 min under physiological conditions and (B) after spinal cord hemisection, in a square open field arena, for both genotypes (CX3CR1 +/+ and CX3CR1 +/eGFP). Analysis of the time spent in the periphery during the 10‐min test under the same conditions (C, physiological condition, and D, SCI). Results are presented as mean ± SEM at each time point for CX3CR1 +/+ females (open black triangles, dashed line) and CX3CR1 +/eGFP females (open green triangles, solid line). Statistics: Two‐way ANOVA followed by Bonferroni post hoc test. Sample size: Uninjured: 2 CX3CR1 +/+ and 6 CX3CR1 +/eGFP mice; SCI: 10 mice per genotype. These mice were used in a previous study (Noristani, They et al. 2018) and reanalyzed for the present work.

## Data Availability

All data included in the article are available from the corresponding author on reasonable request.
