# Supplementary figures and images for: Behavioral Settings are Crucial for Assessing Sensorimotor, Anxiety, and Social Changes in Aging and Spinal Cord Injury
Source: Brain Behav. 2025 Jul 11;15(7):e70686. doi: 10.1002/brb3.70686 (PMC12246551; doi:10.1002/brb3.70686)

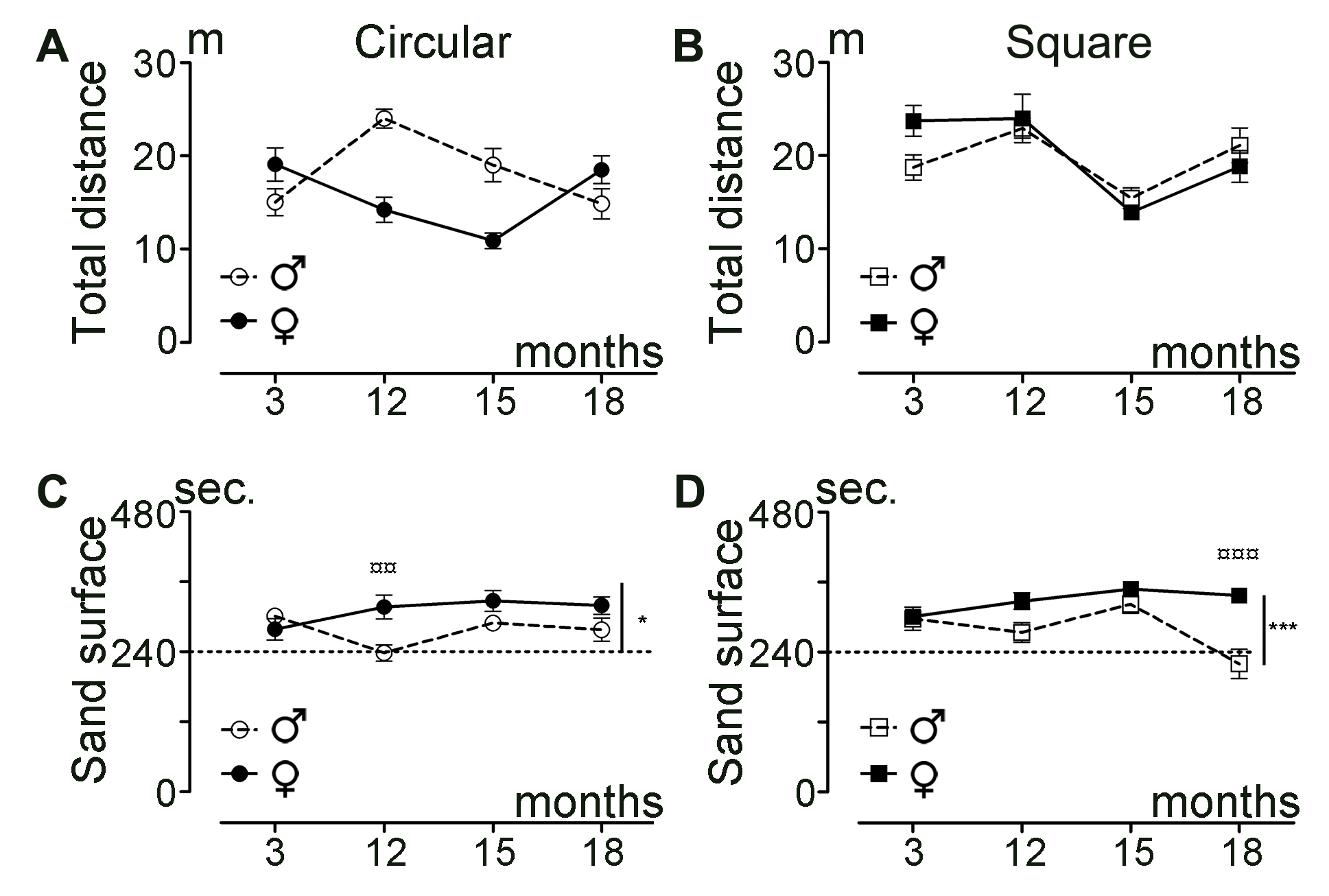

Supplement: Supplementary file 1 — Supporting Figure 1: Sex‐dependent sensorimotor activity in male and female mice over physiological aging. Analysis of the covered distance (A, B) by male and female mice in circular (A) and square (B) open‐field arenas over aging. Analysis of the time spent on the sand surface (C, D) by both sexes in a circular (C) and square (D) arena over aging. Results are presented as mean ± SEM per time point for males (empty circles [A] or squares [B] with dashed lines) and for females (plain circles [A] or squares [B] with solid lines). Statistics: two‐way repeated‐measure analysis of variance (ANOVA) (*p ≤ 0.05; ***p ≤ 0.001), followed by Bonferroni post hoc test (¤¤p ≤ 0.01; ¤¤¤p ≤ 0.001). Number of mice: 13 males and 14 females per experiment and per age. [file BRB3-15-e70686-s003.tif]

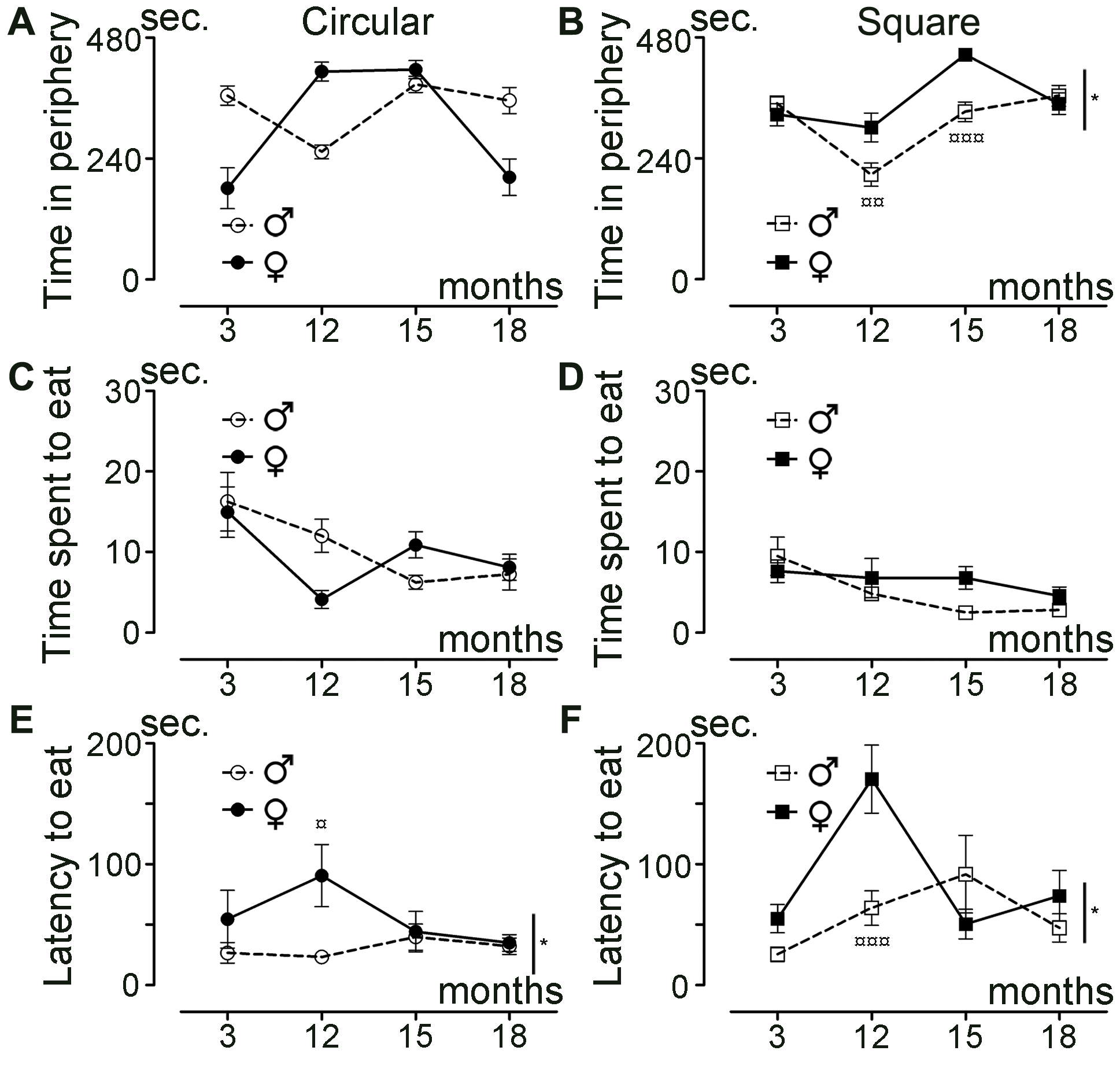

Supplement: Supplementary file 2 — Supporting Figure 2: Sex‐dependent anxiety in male and female mice over physiological aging. Analysis of the time spent in the periphery (A, B) for both sexes in a circular (A) and square (B) open‐field arenas over aging. Analysis of the time spent to eat (C, D) by male and female mice in a circular (C) and square (D) arenas over aging. Analysis of the latency to eat for both sexes in a circular (E) and square (F) arenas over aging. Results are presented as mean ± SEM per time point for males (empty circles (A, C, and E) or squares (B, D, and F) with dashed line) and for females (plain circles (A, C, and E) or squares (B, D, and F) with solid line). Statistics: two‐way ANOVA (*p≤0.05), followed by Bonferroni post hoc test (¤p ≤ 0.05; ¤¤p ≤ 0.01; ¤¤¤p ≤ 0.001). Number of mice: 13 males and 14 females per experiment and per age. [file BRB3-15-e70686-s002.tif]

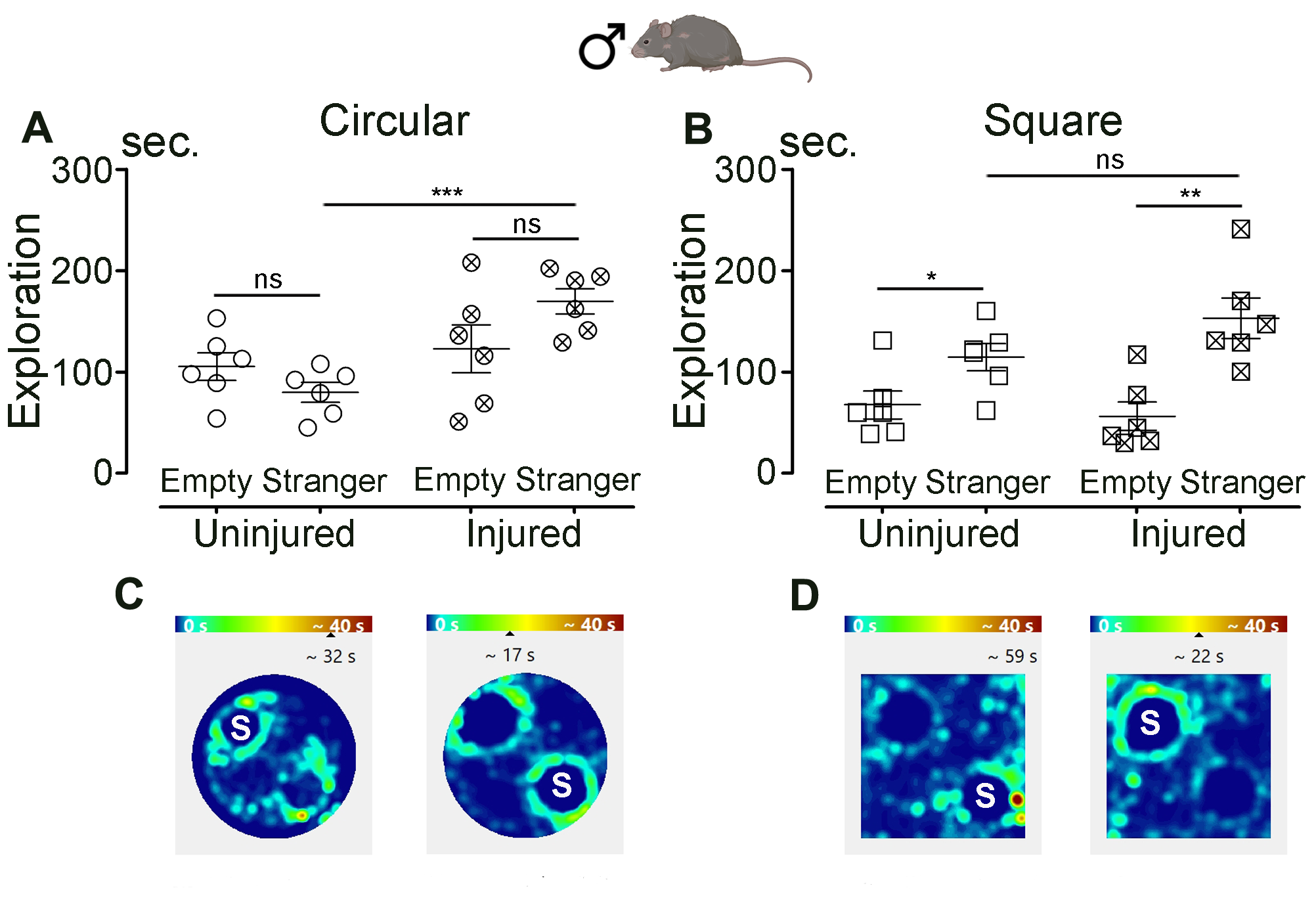

Supplement: Supplementary file 3 — Supporting Figure 3: Influence of the shape of the open field on social behavior in old male in physiological context and at chronic stage after SCI Analysis of the time spent by male mice in the close vicinity of an empty area or a stranger mouse in a circular (A) or square (B) arena at 12 months of age, under physiological conditions, and at 12 months post‐injury (i.e, 15 months of age). Representative heatmaps showing time spent in the circular (C) or square (D) arena during the social interaction test in both experimental conditions. The location of the stranger mouse is indicated by “S” on the heatmaps. Results are presented as mean ± SEM for each arena (uninjured mice are represented with clear circles or squares, and injured animals are represented with cross‐filled symbols). Statistics: Unpaired t‐test with Welch's correction (*p ≤ 0.05; **p ≤ 0.01; ***p ≤ 0.001; ns: nonsignificant). Number of mice: 6 males per group. [file BRB3-15-e70686-s004.tif]

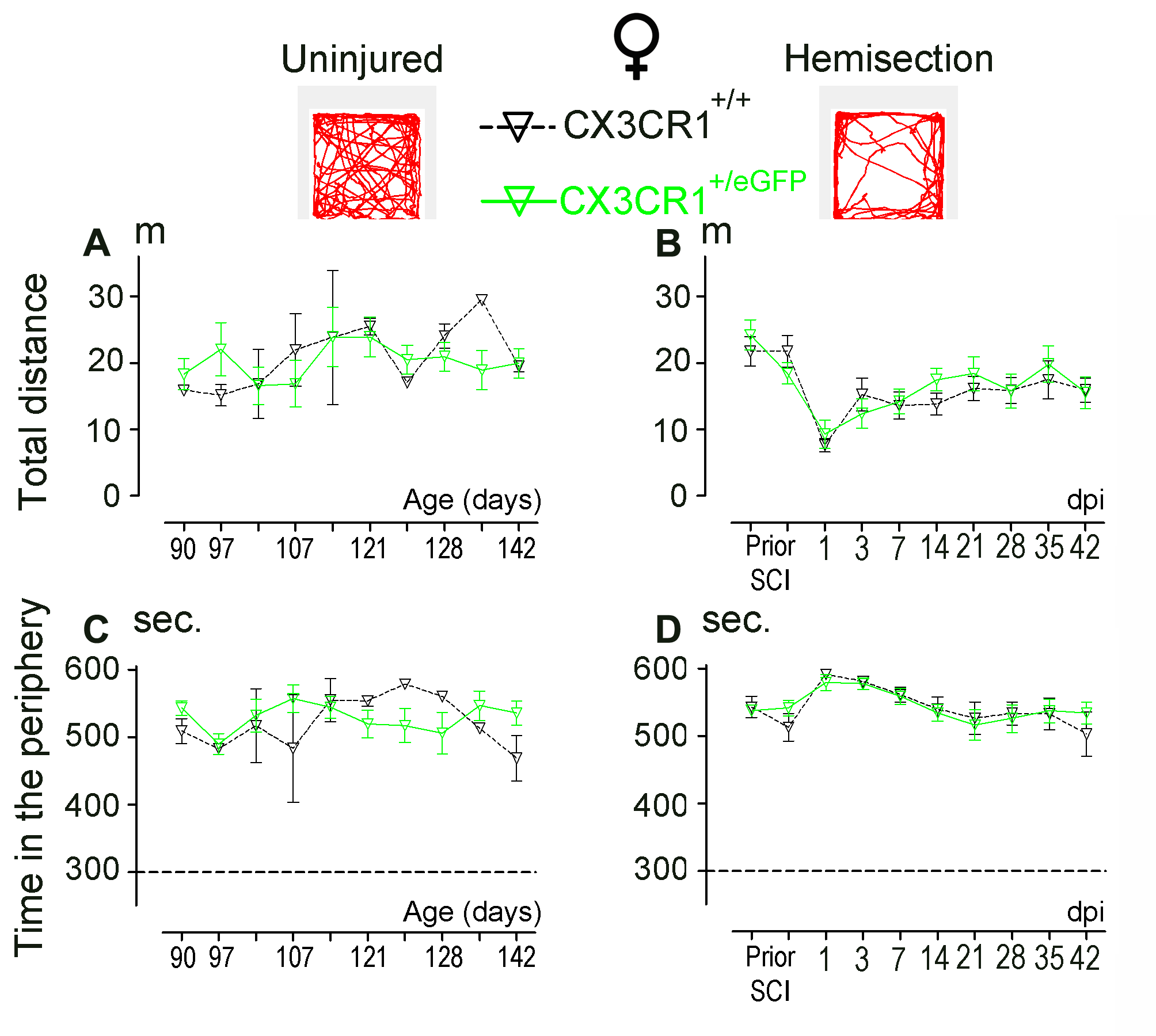

Supplement: Supplementary file 4 — Supporting Figure 4: Female CX3CR1 +/+ and CX3CR1 +/eGFP mice display identical open field motor performance under physiological conditions and SCI (A) Analysis of the distance covered in 10 min under physiological conditions and (B) after spinal cord hemisection, in a square open field arena, for both genotypes (CX3CR1 +/+ and CX3CR1 +/eGFP). Analysis of the time spent in the periphery during the 10‐min test under the same conditions (C, physiological condition, and D, SCI). Results are presented as mean ± SEM at each time point for CX3CR1 +/+ females (open black triangles, dashed line) and CX3CR1 +/eGFP females (open green triangles, solid line). Statistics: Two‐way ANOVA followed by Bonferroni post hoc test. Sample size: Uninjured: 2 CX3CR1 +/+ and 6 CX3CR1 +/eGFP mice; SCI: 10 mice per genotype. These mice were used in a previous study (Noristani, They et al. 2018) and reanalyzed for the present work. [file BRB3-15-e70686-s001.tif]
